# The Role of Pomegranate ( *Punica granatum* ) in Cancer Prevention and Treatment: Modulating Signaling Pathways From Inflammation to Metastasis

**DOI:** 10.1002/fsn3.4674

**Published:** 2025-01-31

**Authors:** Abdur Rauf, Ahmed Olatunde, Zuneera Akram, Hassan A. Hemeg, Abdullah S. M. Aljohani, Waleed Al Abdulmonem, Ahood Khalid, Anees Ahmed Khalil, Md. Rezaul Islam, Rekha Thiruvengadam, Seung‐Hyun Kim, Muthu Thiruvengadam

**Affiliations:** ^1^ Department of Chemistry University of Swabi Anbar Khyber Pakhtunkhwa Pakistan; ^2^ Department of Medical Biochemistry Abubakar Tafawa Balewa University Bauchi Nigeria; ^3^ Department of Pharmacology, Faculty of Pharmaceutical Sciences Baqai Medical University Karachi Pakistan; ^4^ Department of Clinical Laboratory Sciences, College of Applied Medical Sciences Taibah University Al‐Medinah, Al‐Monawara Saudi Arabia; ^5^ Department of Medical Biosciences, College of Veterinary Medicine Qassim University Buraydah Saudi Arabia; ^6^ Department of Pathology, College of Medicine Qassim University Buraydah Saudi Arabia; ^7^ University Institute of Diet and Nutritional Sciences, Faculty of Allied Health Sciences The University of Lahore Punjab Pakistan; ^8^ Department of Pharmacy, Faculty of Allied Health Sciences Daffodil International University Dhaka Bangladesh; ^9^ Center for Global Health Research, Saveetha Medical College, Saveetha Institute of Medical and Technical Sciences (SIMATS) Saveetha University Chennai India; ^10^ Department of Crop Science, College of Sanghuh Life Science Konkuk University Seoul Republic of Korea

**Keywords:** angiogenesis, apoptosis, metastasis, neoplastic, pomegranate

## Abstract

*Punica granatum* , commonly known as pomegranate, is a traditional medicinal agent owing to its antiquity. The scientific literature has shown that pomegranate extracts exhibit favorable modulation of diverse signaling pathways. These pathways encompass those implicated in inflammation, angiogenesis, hyperproliferation, cellular transformation, tumorigenesis initiation, and ultimately, a reduction in advanced metastasis and tumorigenesis. Pomegranate extracts in this context can be attributed to their high polyphenol content, which has been observed to possess inhibitory properties toward specific signaling pathways associated with cancer. As a formidable pathology, cancer is the most significant cause of death worldwide after cardiovascular disease. The annual incidence of cancer‐related mortality has increased progressively. Modifying one's dietary patterns, engaging in regular physical exercise, and maintaining an optimal body mass index are three straightforward measures that an individual may undertake to mitigate their susceptibility to cancer. Incorporating diverse vegetables and fruits into one's dietary regimen exhibits promising potential for preventing a minimum of 20% cancer incidence and approximately 200,000 cancer‐related mortalities annually. Vegetables and fruits contain high levels of minerals and phytochemicals, which help alleviate and prevent the harmful effects of cancer. These substances are safe and exhibit minimal toxicity in biological systems. Furthermore, they exhibit antioxidant properties and have garnered extensive approval for their use as nutritional supplements. Pomegranates are used in ancient cultures to prevent and treat various diseases. Extensive research on pomegranate extract, fruit, oil, and juice has revealed promising findings regarding their potential anti‐proliferative, anti‐tumorigenic, and anti‐inflammatory properties through the modification of various signaling pathways related to cancer, thus demonstrating their potential as drugs to prevent and treat cancer. Emerging research indicates that pomegranate can potentially prevent and treat different cancers, including prostate, bladder, breast, skin, lung, and colon cancer.

## Graphical abstract

*Punica granatum* (pomegranate) exhibits significant anti‐cancer properties due to its polyphenol‐rich extracts, which modulate signaling pathways involved in inflammation, angiogenesis, and tumor progression. Review highlights its potential to prevent and treat various cancers, including prostate, breast, lung, and colon cancers, by leveraging its antiproliferative and anti‐inflammatory effects. Pomegranate's safe and antioxidant‐rich phytochemicals make it a promising dietary supplement for cancer prevention and treatment, aligning with broader dietary strategies to reduce cancer incidence and mortality.

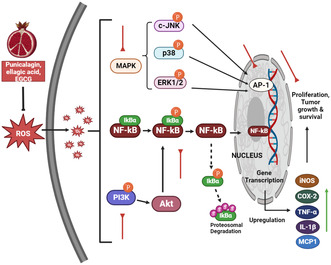

## 1 Introduction

Pomegranates ( *Punica granatum* L.) are predominantly found in Asian countries. In ancient Egypt, the fruit was utilized for sarcophagi decoration and was seen as an icon of prosperity and ambition (Mars 2000; Bassiri‐Jahromi 2018; Moga et al. 2021). In Ayurvedic tradition, the fur of the pomegranate fruit has been regarded as a comprehensive source of medicinal compounds, offering a wide range of health benefits (Naqvi, Khan, and Vohora 1991). Pomegranate fruits and trees are grouped into many compartments, including the peel, juice, root, leaf, bark, and flower, and each component provides secondary compounds with therapeutic properties. Peels and juices have been reported to exhibit potent antioxidant activities. In addition, juice and oil are slightly estrogenic and can be used to treat postmenopausal manifestations (Lansky and Newman 2007). The pomegranate fruit contains numerous seeds in a white membrane coated with pericarps, accounting for 50% of its weight. The pomegranate fruit contained 50% seeds in a white membrane that was covered with pericarps. The seeds contained 20% oil and had 10% fruit's weight (Viuda‐Martos, Fernández‐López, and Pérez‐Álvarez 2010). Bioactive substances, such as quercetin, kaempferol, luteolin, ellagitannins, and anthocyanidins, are abundant in pomegranate (Ben Nasr, Ayed, and Metche 1996; Noda et al. 2002; Mertens‐Talcott et al. 2006).

Pomegranate is a fruit rich in secondary metabolites (Miguel, Neves, and Antunes 2010), and its various products, particularly juice, have gained popularity because of their bioactive composition, particularly polyphenols (Hegazi et al. 2021; Kalaycıoğlu and Erim 2017). The primary polyphenols found in this plant include hydroxybenzoic acids, hydroxycinnamic acids, anthocyanins, flavonoids, proanthocyanidins, leucoanthocyanidins, and hydrolyzable and condensed tannins (Kohno and Kokkinomagoulos 2020). Other vital phenolics with health‐promoting properties include punicalin (α and β), punicalagin α and β garantin (A and B), and pedunculagin (Mathon et al. 2019), all of which are found in pomegranate. Compounds found in pomegranate have been reported to exhibit various health‐promoting effects, including decreased erythropoietin levels in the serum of patients with type 2 diabetes, reduction in the levels of diastolic and systolic pressure, low‐density lipoprotein (LDL) and total cholesterol (Sohrab et al. 2019), reduction in creatinine level, blood pressure, C‐reactive protein, and muscle damage in middle‐aged athletic individuals (Achraf et al. 2018). Furthermore, pomegranate compounds decreased LDL, triglyceride, total cholesterol, and glucose levels in overweight individuals. Punicalagin (α and β forms) has been reported to exhibit anti‐proliferative action in lung and prostate cancer cell lines (Kohno and Kokkinomagoulos 2020).

## 2 Composition of Pomegranate

### 2.1 Primary Components in Pomegranate

The oils found in the pomegranates are rich in fatty acids. The fatty acid composition of this plant exceeds 95% of its oil content. Within this composition, approximately 99% of the fatty acids present are in the form of triglycerides. Minor oils are composed of steroids, sterols, and a significant composition of cerebroside and myelin sheaths (Kohno et al. 2004; Lansky and Newman 2007). Intriguingly, pinicic acid is a conjugated derivative found in the oil of pomegranate and constitutes approximately 70%–76% of the seed oil (Viladomiu et al. 2013). In pomegranate seeds, amino acids such as leucine and phenylalanine were identified. This study discovered nucleosides such as adenosine, crotonoside, and guanosine (Li et al. 2020). The pericarps of the pomegranates consist of minerals and complex polysaccharides. These minerals include nitrogen, Na, Mg, phosphorus, Ca, and potassium (Viuda‐Martos, Fernández‐López, and Pérez‐Álvarez 2010). Pomegranate juice contains trace amounts of fatty acids, organic acids, and minerals such as Zn, Se, Ca Co, Mg, and Cs at medium concentrations (Waheed et al. 2004). The seed oil of pomegranate contains fatty acids, which account for more than 95% of its oil content (Lansky and Newman 2007). Punicic acid, an isomer of linoleic acid, is predominant in the seeds of plants and accounts for approximately 76% of seed oil (Kohno et al. 2004). Other trace elements include steroids, sterols, and cerebrosides, but the seed coat contains organic acids, such as ascorbic acid, vitamin C, and malate (Elfalleh et al. 2012). Pomegranate leaves contain minerals, such as calcium, iron, sodium, and potassium. The young leaves of pomegranate have high potassium levels, but the mature leaves contain more iron and calcium. Medium‐aged plant leaves have been reported to contain high levels of sodium (Zhang et al. 2010). Polyholoside (2.58%), another vital bioactive compound, is found in the rind of the pomegranate fruit. Sugars such as fructose, raffinose, and glucose are also present in low amounts, whereas pectic substances, water‐soluble polyholosides, and hemicelluloses A and B are also present in pomegranates (Keogh and O'Donovan 1970; Jurkovic, Mikelic, and Smit 1976). Most of these sugars, including glucose, mannose, galacturonic acid, rhamnose, and galactose, have been isolated from pomegranate fruit (Fadavi et al. 2005).

### 2.2 Major Secondary Components

The chemical constituents of pomegranates differ depending on the growing region, climate, storage conditions, agricultural method, cultivar, and maturity (Guo et al. 2008). Half of a fruit's weight can be found in its peel, which contains secondary metabolites such as proanthocyanidins, phenolics, flavonoids, polysaccharides, and ellagitannins. Approximately 50% of the edible portion of the pomegranate fruit contains 10% seeds and 40% arils. The arils contain pectin at a concentration of 1.5%, sugars at 10% (primarily glucose and fructose), water at 85%, organic acids including malic acid, citric acid, vitamin C (ascorbic acid), and other secondary metabolites such as flavonoids and phenolic compounds (Viuda‐Martos, Fernández‐López, and Pérez‐Álvarez 2010). The fruit seeds contain cyanidin‐3‐glucoside, pelargonidin‐3,5‐diglucoside, cyanidin‐3,5‐diglucoside, delphinidin‐3‐glucoside, delphinidin‐3,5‐diglucoside, and pelargonidin‐3‐glucoside. Among the various anthocyanins present, delphinidin‐3,5‐diglucoside exhibits the highest prevalence in pomegranates (Elfalleh et al. 2012). The total seed weight (12%–20%) consisted of seed oil containing > 70% conjugated linolenic acids. Pomegranate skin is rich in pelargonidin‐3‐glucoside and pelargonidin‐3,5‐diglucoside, whereas pomegranate arils contain only trace levels of these compounds. Both the fruit rind and arils contained cyanidin‐3,5‐diglucoside and cyanidin‐3‐glucoside, respectively. The pomegranate fruit rinds, on the other hand, did not contain any detectable levels of delfinidin‐3‐glucoside or delfinidin‐3,5‐diglucoside (Hartwell 1971; Jain et al. 2014).

Pomegranates contain various natural antioxidants including flavonoids, anthocyanins, phenolic compounds, and tannins. These secondary metabolites are found in multiple parts of the fruit (Table 1). This may explain the claimed superiority of the blended form of the fruit over isolated pomegranate extracts (Seeram and Nair 2002). A comparative study indicated that anthocyanins in pomegranate fruit possess greater antioxidant activity than vitamin C and α‐tocopherol (Vidal et al. 2003). Phenolic acids include 4‐hydroxycinnamic acid, brevifolincarboxylic acid, ferulic acid, sinapic acid, ellagic acid, cinnamic acid, isoferulic acid, ursonic acid, maslinic acid, corosolic acid, oleanonic acid, salicylic acid, punicalin, caffeic acid, and betulonic acid (Li et al. 2020).

**TABLE 1:** Secondary metabolites in various pomegranate parts.

Part of pomegranate	Secondary metabolites	References
Pomegranate peel	Caffeic acid, quercetin, gallic acid, ellagitannins, punicalin, pelletierine, luteolin, ellagic acid, and kaempferol	(Brieskorn and Keskin 1954; Tanaka, Nonaka, and Nishioka 1986; Gil et al. 2000; Neuhöfer et al. 1993; Van Elswijk et al. 2004; Nawwar, Hussein, and Merfort 1994)
Pomegranate bark and root	Piperidine, Pelletierine, and pyrrolidine alkaloids	(Gil et al. 2000; Neuhöfer et al. 1993; Van Elswijk et al. 2004)
Pomegranate leaf	Sterols, reducing sugars, piperidine alkaloids, carbohydrates, flavonoids, tannins, flavone, glycoside, and ellagitannins	(Gil et al. 2000; Chaitra et al. 2012; Cui et al. 2004)
Pomegranate juice	Aliphatic organic acids, gallic acids, ellagic acids, simple sugars, ascorbic acid, amino acids, quinic acid, and flavonols	(Poyrazoğlu, Gökmen, and Artιk 2002; Nawwar, Hussein, and Merfort 1994; Tanaka, Nonaka, and Nishioka 1986; Drillien and Viel 1963; Lansky and Newman 2007; Wang et al. 2004)
Pomegranate seed	3,3′‐Di‐O‐methylellagic acid, oleic acid, 3,3′,4′‐Tri‐O‐methylellagic acid palmitic acid, stearic acid, punicic acid, tocopherols, sterols, linoleic acid, and sex steroids	(Schubert, Lansky, and Neeman 1999; Hornung, Pernstich, and Feussner 2002; Wahab et al. 1998; Kim et al. 2002; Lansky et al. 2005; Huang et al. 2005)
Pomegranate flower	Fatty acids, gallic acids, ursolic acid, triterpenoids	(Batta and Rangaswami 1973; Lan et al. 2009; Zelano et al. 2022)

Li et al. (2020) identified secondary metabolites in pomegranate seeds using ultra‐high‐performance liquid chromatography (UHPLC) linked to quadrupole Orbitrap high‐resolution mass spectrometry. In this study, 88 chemical molecules, including flavonoids, phenolic acids, and coumarins, were tentatively identified in seeds. Flavonoids include rutin, luteolin, apigenin, genistein, scutellarein, kaempferol, gallocatechin and dihydromyricetin. Coumarin was identified in pomegranate seeds using UHPLC‐Q‐Orbitrap HRMS. These include umbelliferone, 7‐methoxycoumarin, scoparone, and 4‐methylumbelliferone (Li et al. 2020). Alkaloids, including pelletierine, were found to be an integral part of the pomegranate peel. However, their presence remains speculative (Brieskorn and Keskin 1954). Phytochemicals represent about 0.35%–0.60% in fruit and more than 3% in pomegranate roots. However, no effect was observed on the fruit rind (Cáceres et al. 1987; Chidambara Murthy, Jayaprakasha, and Singh 2002). The alkaloids include pseudopelletierine, isopelletierine, pelletierine, and methyl isopelletierine. Dipelletierine and methylpelletierine 1‐pelletierine were discovered in the rinds and roots of pomegranates (Dean, Exley, and Goodwin 1971; Du, Wang, and Francis 1975). Pomegranate leaf extract contains 2‐(2‐propenyl)‐piperidine, an unsaturated alkaloid, but not saturated alkaloids in the body rinds and roots (Drillien and Viel 1963).

Tannins and their related molecules have also been identified in pomegranates. Novel ellagitannins, called punicacorteins D, C, B, and A, with hydrolyzable C‐glycoside structures, have been found in pomegranate roots. In addition, the plant contains gluconic acid‐containing compounds casuariline and casuarina (Fayez, Negm, and Sharaf 1963; Feldman and Markh 1970). Punicafolin, a compound belonging to the class of ellagitannins, four other ellagitannins, and two gallotannins have been identified in pomegranate leaves. The compounds identified in this study were granatin B and A, corilagin, strictinin, 1,2,3,4,6‐penta‐O‐galloyl D‐glucose, and 1,2,4,6‐tetra‐O‐galloyl D‐glucose (Isamuhamedov and Akramov 1982). Triterpenic acids such as ursolic acid have been reported to represent 0.45% in flowers and leaves, and 0.6% in pomegranate fruits (Amakura et al. 2000) (Table 1).

## 3 Pomegranate‐Specific Oncogenic Signaling Pathways

Cancer continues to be widely acknowledged as a highly perilous and formidable ailment despite the numerous advancements in diagnostic techniques and therapeutic approaches that have been devised (Marrie et al. 2022). Credible methods for controlling cancer incidence and progression include establishing early intervention techniques, using effective treatments for indigenously metastatic melanoma, and applying adequate management protocols for cancers that have spread beyond their original site. In the long term, these steps reduce the cancer‐related mortality and morbidity rates. Additional alternatives include detection and treatment at an early stage, streamlined cancer care transcending geographical boundaries, and efficacious systemic cancer therapeutics with non‐specific targeting capabilities. Cancer prevention seems to be the best way to reduce its prevalence and impact (Boeing et al. 2012). Chemoprevention involves the utilization of both naturally occurring and artificial bioactive compounds to exert inhibitory, arresting, reversing, or retarding effects on carcinogenesis (Rahman et al. 2022). Scientific evidence has established a strong association between a specific dietary pattern characterized by substantial fruit and vegetable consumption and a noteworthy decrease in the likelihood of developing and advancing cancer (Seeram et al. 2005). Epidemiological studies have supported this correlation extensively. These foods contain phytochemicals, such as isoflavones, flavonoids, minerals, vitamins, lycopene, and fiber. All these factors aid in achieving optimal health and warning of diseases, including cancer (Jurenka 2008). The structural composition, quantity, and physiological role of antioxidants in fruits and vegetables are widely acknowledged. Oxidative stress can manifest in the human body and affect antioxidant functionality (Habib et al. 2023).

The scientific community has conducted significant research on the therapeutic potential of pomegranate, as evidenced by studies on its health advantages conducted and published in recent decades (Lansky and Newman 2007). Pomegranate is used by the Unani Medical System to treat cancer. Following this practice, pomegranate exhibits properties that imply its effectiveness in cancer treatment (Syed, Afaq, and Mukhtar 2007). The pomegranate fruit and its bark, leaves, and roots contain high concentrations of molecular components with therapeutic potential. Recent findings have revealed that pomegranate and its constituent elements can significantly modify numerous signaling pathways linked to inflammation, cellular transformation, hyperproliferation, angiogenesis, oncogenesis, and metastasis (Zaid et al. 2007). The constituents of pomegranate fruit have been shown to exhibit regulatory effects on pro‐inflammatory mediators, growth factors, anti‐apoptotic proteins, and cell adhesion molecules. Scientists have studied galactomannan isolated from fruit rinds and pomegranate extracts. They focused on the effects of this substance on skin cells, specifically targeting the activation of the STAT3, MAPK (UVB‐mediated), and NF‐κB signaling pathways. This investigation encompassed the synergistic integration of in vivo and in vitro analyses. The scientists observed a down‐regulation of VEGF and MMPs pathways, which contributed to the anti‐tumor activity of this substance (Afaq et al. 2008; Hosseini et al. 2011; Varghese et al. 2017; Rahman et al. 2023). In a comprehensive review, Rahman et al. (2023) elucidated the intricate molecular targets of pomegranate in breast cancer using a cellular targeting approach. This study demonstrates anti‐aromatase and anti‐estrogenic properties and concurrently diminishes pro‐inflammatory cytokines, chemokines, and VEGF. Rettig et al. (2008) studied the anti‐proliferative properties of pomegranate extract in androgen‐independent prostate cancer. Wang et al. (2012) demonstrated that the inhibition of cancer proliferation is facilitated by a mechanism reliant on NF‐κB. This study examined the potential inhibitory effects of specific components of pomegranate juice, namely punicic acid, ellagic acid, and luteolin on prostate cancer metastasis (Wang et al. 2012). In an in vivo study, researchers observed that the combined presence of these three components (punicic acid, ellagic acid, and luteolin) inhibited IGF‐1/Akt/mTOR signaling. This inhibition was determined to be the primary mechanism for the inhibition of the proliferation of hormone‐dependent and hormone‐refractory prostate cancer cells. It also hinders their ability to migrate and respond to stromal cell‐derived factor 1α (SDF1α) (Li et al. 2016).

Furthermore, these components have been observed to enhance the expression of cell adhesion genes, while simultaneously reducing the expression of genes associated with cell cycle regulation and migration. In addition, they have been observed to enhance the expression of various well‐established tumor‐suppressive microRNAs (miRNAs), reduce the levels of several oncogenic miRNAs, and impede chemotaxis involving chemokine receptor type 4 (CXCR4) and SDF1α (Li et al. 2016). Li et al. (2020) conducted in vitro experiments using pomegranate leaf extract. Researchers have observed that the extract induces apoptosis through the intrinsic mitochondrial pathway and inhibits migration and invasion in non‐small cell lung cancer (Amin et al. 2009). The compounds found in pomegranates display anticancer activity by influencing vital signaling cascades. For instance, galactomannan from plant extracts stimulates STAT3, MAPK, and NF‐κB cascades, resulting in the inhibition of cancer cell activity. In addition, these compounds also confer antiproliferative activity through the suppression of VEGF, MMPs, and IGF‐1/Akt/mTOR signaling pathways. Hence, inhibition of these vital signaling cascades leads to the death of cancer cells.

## 4 Anti‐Cancer Mechanism of Pomegranate‐Specific Phytochemicals

According to the research conducted by Amin et al., certain constituents of pomegranate possess advantageous properties, such as anti‐proliferative and antioxidant actions (Amin et al. 2009). These properties include cell cycle disruption, growth inhibition, and apoptosis, which are potentially valuable for the treatment of lung, skin, and breast cancers (Shaikh and Bhandary 2021; Hanahan and Weinberg 2011). The beneficial characteristics of pomegranate fruit, juice, seeds, and seed oil have been extensively reported in scientific literature. Researchers have identified several features commonly observed across many types of neoplasia, despite the vast intricacy and heterogeneity of cancer diseases. According to Hanahan and Weinberg, a comprehensive list of cancer hallmarks includes replicative immortality, extended proliferative signaling, apoptosis resistance, invasion, cellular energetics deregulation, angiogenesis, metastasis, immune destruction, and evasion of growth suppressors (Hanahan and Weinberg 2000; Senga and Grose 2021). These characteristics can be ascribed to genomic instability, which accelerates the rate of mutations and inflammation, thereby promoting tumor growth. Senga and Grose further elucidated the pathogenesis of cancer by introducing four features that expanded this concept. These features include epigenetic dysregulation, dedifferentiation (or transdifferentiation), modified microbiota, and altered neuronal signaling (Seeram et al. 2007). Researchers have discovered that pomegranate fruit has potent anti‐cancer effects, effectively inhibiting cancer development in various cancerous tumors in animal models. This approach induces apoptosis and cell cycle arrest in cancer cells by blocking several signaling pathways within cancer cells (Laudisi et al. 2018). Jak/STAT, NF‐B, and MAPK/ERK are only a few of the numerous signaling pathways utilized by cancer cells. Differentiation, proliferation, angiogenesis, and metastasis are hallmarks of cancer cells, which often exhibit constant overactivation or downregulation. Multiplying these signaling pathways with synthetic or natural drugs provides a rationale for cancer prevention and treatment.

### 4.1 STAT3 Pathway and Its Modulation

In the context of proteins, “STAT” refers to a family of enzymes that functions as signal transducers and transcriptional activators. Dormant intracellular and cytosolic transcription factors constitute this class of proteins. Transcription factors play pivotal roles as mediators of cellular response to endogenous and extrinsic stimuli. Many important biological genes involved in inflammation, cell proliferation, angiogenesis, and apoptosis are either expressed or suppressed in response to activating proteins (Sgrignani et al. 2018; Uddin et al. 2022). Seven distinct human protein subtypes belong to the STAT family. STAT1, STAT2, STAT3, STAT4, STAT5a and b, and STAT6 are abbreviations for the following family members: STAT3 encodes the widely recognized oncogenic protein, STAT3. Its action is regulated by several cellular mechanisms, including the acetylation, phosphorylation, and redox pathways (Linher‐Melville and Singh 2017; Busker et al. 2020). Nevertheless, it has been observed that while healthy cells can function normally without an active *STAT3* gene (Lau et al. 2019), malignant cells cannot. For cancer cells to multiply, the STAT3 protein must first be activated. STAT3 activation is regulated in normal cells. However, in cancer cells, there is an abnormal dysregulation of STAT3 activation due to increased STAT3 gene expression.

Consequently, cancer cells undergo dysregulation of various biological processes including angiogenesis, autophagy, differentiation, cell proliferation, metabolic alterations, immunosuppression, apoptosis, and cell survival (Heber 2008; Lau et al. 2019). The scientific literature has elucidated that certain molecular entities that fall under *Punica granatum* ‐specific phytochemicals manifest as onco‐protective or onco‐preventive attributes (Weng and Yen 2012; Avalle et al. 2017). Extensive studies have been conducted on various phytochemicals, particularly pomegranates, with respect to multiple types of cancers. The phytochemicals under investigation have been postulated to possess the capability and specialized ability to impede unregulated STAT3 signaling pathways (McCormick, Chu, and Vermeren 2019; Sarwar et al. 2020; Aziz et al. 2021). Consequently, identifying novel medicines with enhanced safety and efficacy is a formidable endeavor. This review examines the phytochemical substances found in pomegranates that specifically affect STAT3 and its target genes, to explore their potential for cancer treatment.

## 5 Pomegranates and the PI3K/Akt Signal Transduction Pathway

Extensive research has been conducted on anti‐cancer therapies, emphasizing the phosphoinositide 3‐kinase/ protein kinase B (PI3K/Akt) pathway. The present prototype survival pathway additionally receives extrinsic signals from numerous membrane receptors, including EGFR, and assumes a pivotal function in cellular survival and angiogenesis through diverse mechanisms. Glycolytic phenotypes have been observed in most cancer types. The glycolytic phenotype observed in cancer cells is often attributed to the PI3K pathway, which is linked to the serine/threonine kinase, Akt. Phosphoinositide 3‐kinases (PI3Ks) are a discrete family of kinases comprising of three subtypes. Phosphorylation of phosphatidylinositol 4,5 bisphosphate (PIP2) is the most important subtype. The outcome was production of phosphatidylinositol 3‐phosphate (PIP3).

Consequently, alterations in the activity of subsequent proteins are regulated by intracellular PIP3 (Yu and Cui 2016; Hao et al. 2017). These proteins interact with a wide range of membrane receptors. Therefore, this elucidates its significance in several pathological conditions such as cancer (Martini et al. 2014). Strategically focusing on the PI3K pathway in cancer therapy is motivated by the importance of PI3K signaling as a significant mechanism in cancer progression. The motivation for this research stems from studies that have demonstrated the prominent involvement of PI3K signaling as the predominant pathway associated with cancer. As previously stated, the PI3K signaling pathway can affect every stage through carcinogenic processes.

In addition, the activation of this pathway has been shown to serve as a signal for determining prognosis and predicting chemotherapy efficacy in patients (Yang et al. 2019). However, pursuing this trajectory is not a simple endeavor but rather a fraught with challenges, as evidenced by many research findings. However, several limitations of PI3K targeting have been identified. Notably, PI3K targeting rarely leads to mortality. By inhibiting PI3K signaling, cancer cells have established inherent methods to evade treatment. Third, the observed inhibition of PI3K was not consistent with the anticipated therapeutic outcomes. Finally, treatment with PI3K has been associated with increased insulin levels owing to its effect on metabolism, resulting in hyperglycemia and other related complications (Fruman and Rommel 2014).

Six categories of pharmacological agents effectively inhibit the PI3K signaling system, each exhibiting a distinct mechanism of action (Mirza‐Aghazadeh‐Attari et al. 2020; Wang 2016). Inhibitors of the mammalian target of rapamycin (mTOR) kinase, dual inhibitors of PI3K and mTOR, and inhibitors of the protein kinase Akt are all included in the drug classes. Akt, PDK1, and mTOR are only a few PI3K signaling molecules that polyphenols can affect (Li et al. 2018; Khan et al. 2012). Many cancers are characterized by aberrant phosphoinositide‐3‐kinase (PI3K) and Akt expression. Evidence from previous studies suggests that drinking pomegranate juice may suppress the mRNA expression of NF‐B and VCAM‐1.

Moreover, it enhanced miR‐126 expression and concomitantly reduced mTOR and PI3K phosphorylation. The efficacy of pomegranate juice consumption in reversing these effects has been demonstrated previously. Based on the findings of an additional investigation, it has been observed that pomegranate juice can impede the activation of TNF‐α, a pivotal protein essential for the appropriate modulation of the NF‐κB transcription factor. Recent studies have examined the effects of pomegranate juice and its extracts on two prostate cancer cell lines: PC‐3 and DU‐145. The main phenolic components of the peel extract were ellagic acid and punicalagin. These results indicate that pomegranate peel extract alters the mTOR/S6K signaling pathway in prostate cancer cells.

**FIGURE 1:**
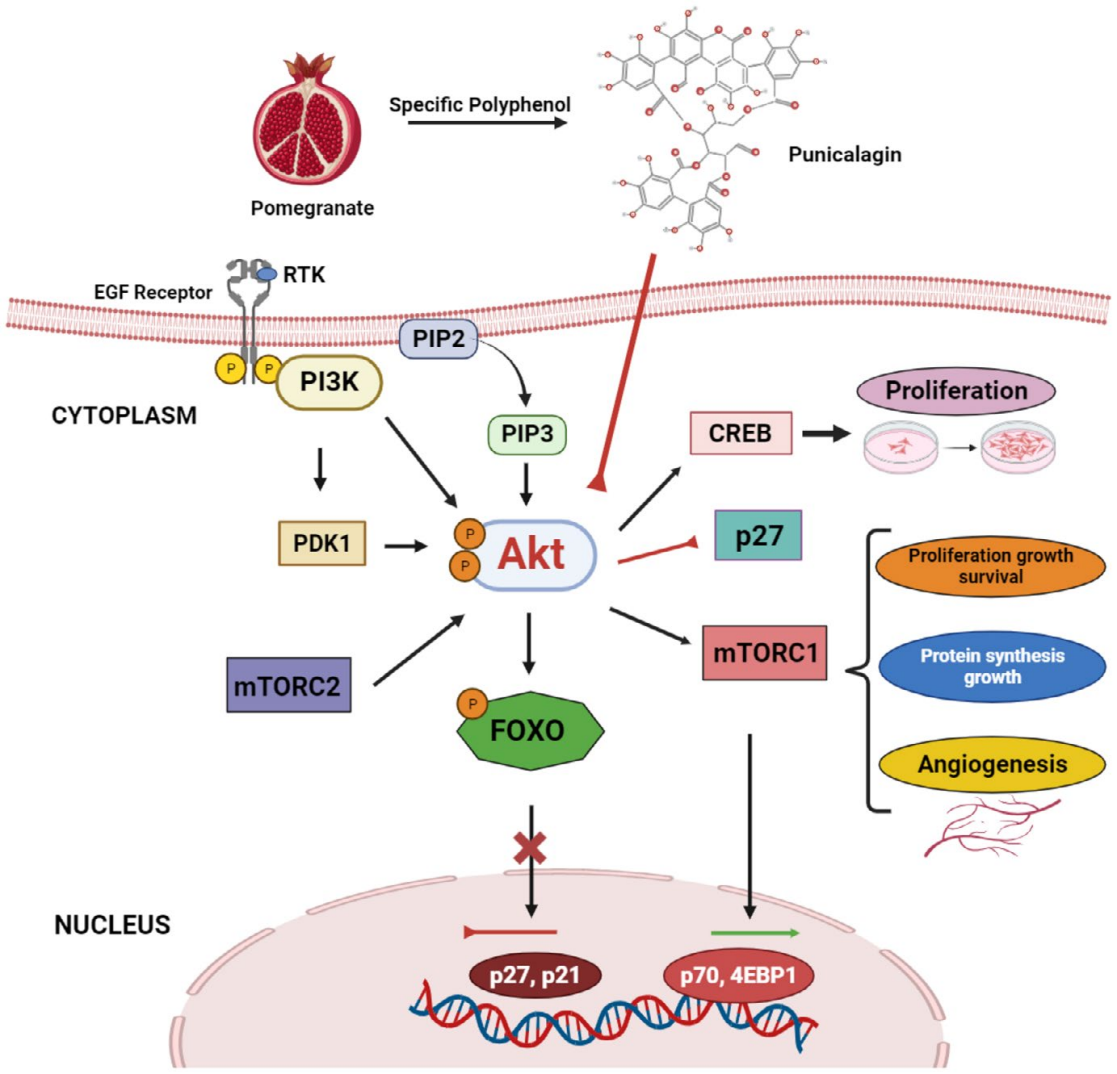
Effect of pomegranate specific polyphenols on PI3K/Akt Signal Transduction Pathway.

Consequently, it exerts a robust antineoplastic effect on cellular milieu. Previous studies have demonstrated the potential of ellagic acid, a metabolite derived from punicalagin, in exerting chemopreventive effects against carcinogenesis. A comprehensive study involving cervical cancer showed that administering a therapy containing 2.5 μM ellagic acid impaired the Akt/mTOR signaling pathway (Figure 1). The upregulation of IGFBP7 expression was implemented to attain the desired outcome, potentially impeding the infiltration of HeLa cells into tissue (Rettig et al. 2008). In addition, punicalagin from pomegranate inhibited the Akt signaling cascade, including suppression of Forkhead Box class O (FOXO) and decreased expression of p27 and p21 genes. In addition, the compound stimulates the mTORC1 and CREB signaling cascades and upregulates the p70 gene. Thus, punicalagin induced cancer cell proliferation via these mechanisms (Figure 1).

## 6 Pomegranates and the NF‐κB and MAPK Signal Transduction Pathway

**FIGURE 2:**
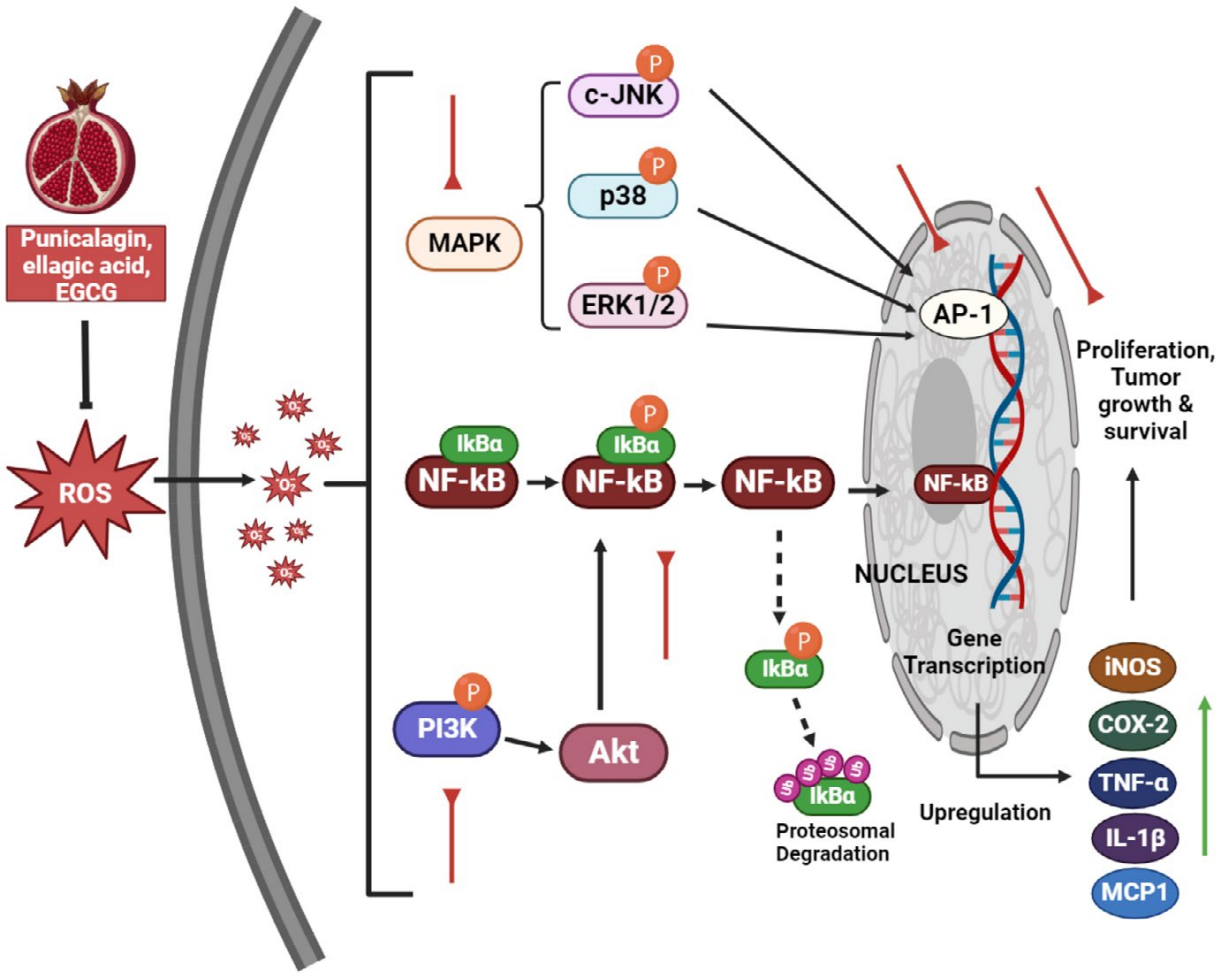
Effect of pomegranate specific polyphenols on NF‐κB and MAPK Signal Transduction Pathway.

MAPKs, or mitogen‐activated protein kinases, are a class of enzymes that primarily phosphorylate serine and threonine residues. These kinases are essential for controlling the activation of transcription factors NF‐B and AP‐1 in response to environmental stimuli. p38 kinase, c‐Jun N‐terminal kinase, and extracellular signal‐regulated kinase 1 and 2 (ERK1/2) are members of the MAPK family (Chen et al. 2001; Hart and DeMarco 2008). Several members of the MAPK family, such as p38 and ERK1/2, have been shown to modulate NF‐κB activation via the IKK‐NF‐B pathway (Ben‐Neriah and Karin 2011). NF‐κB, a transcription factor, modulates various cellular functions including cellular stress, proliferation, survival, and inflammation (Mix et al. 2001; Larrosa et al. 2010). The administration of punicalagin resulted in a notable decrease in p‐JNK levels, suggesting an inhibitory effect of punicalagin on the activation of the MAPK pathway (Figure 2). Punicalagin exerts anti‐inflammatory effects on bovines.

To investigate the potential effect of punicalagin on bovine endometritis, we stimulated bovine endometrial epithelial cells with lipopolysaccharide (LPS). Administration of punicalagin prior to the experiment resulted in a significant decrease in the synthesis of IL‐1β, IL‐6, and IL‐8, as indicated in previous findings (Ben‐Neriah and Karin 2011). Punicalagin reduced the phosphorylation of c‐JNK, p38, and ERK during mechanistic molecular investigations, thereby indicating its potential to impede LPS‐induced MAPK induced by LPS (Mix et al. 2001; Larrosa et al. 2010). Based on the findings of this study, pomegranate fruit can effectively inhibit the p38‐MAPK pathway and NF‐κB transcription factor (Karin et al. 2002; Rahimi, Arastoo, and Ostad 2012). The activation of NF‐κB and p38‐MAPK is positively correlated with the upregulation of crucial inflammatory mediators, including COX‐2, IL‐1, TNF‐α, iNOS, and MCP1, in terms of gene expression (Khan et al. 2007). The observed upregulation in gene expression appears to be intricately linked to the activation of the p38‐MAPK signaling pathway. The observed upregulation of gene expression appears to be associated with heightened p38‐MAPK activity. These chemical compounds have been reported to enhance gene expression in various studies. Inhibitory effects of punicalagin on NF‐κB pathway activity in ME‐180 cervical cancer cells were observed. Punicalagin exhibits notable antineoplastic properties in papillary thyroid carcinoma, and is widely recognized as a prevailing endocrine malignancy. In additional investigations on papillary thyroid cancer, punicalagin exhibited the ability to phosphorylate p65, leading to its subsequent destruction and translocation into the nucleus. The available data indicate that punicalagin exerts regulatory effects on the NF‐κB signaling cascade. The hepatoprotective effects of pomegranate polyphenols, specifically punicalagin and ellagic acid, have been observed to mitigate tumor‐induced hepatic injury and apoptosis in rats with tumor burden, as reported by Adams et al. (2006). Modulation of Nrf2 and NF‐κB results in a notable reduction in hepatic injury and apoptotic cell death induced by the neoplasm. One family of transcription factors, NF‐κB, exerts regulatory control over genes implicated in many physiological processes including differentiation, apoptosis, proliferation, and inflammation. This class of transcription factors exhibits gene expression patterns that are important for numerous physiological functions (Adams et al. 2006). According to a previous study, NF‐κB activation or overexpression is associated with various types of cancers. In vitro research has indicated that administering pomegranate extract to prostate cancer cell lines results in a significant decrease in NF‐κB levels and a concurrent increase in apoptosis. The proliferation of lung cancer cells was inhibited by treatment with pomegranate fruit extract, leading to cell cycle arrest in the G0‐G1 phase. Furthermore, the investigation revealed that the administration of the extract reduced NF‐κB binding to DNA within the cellular milieu (Malik et al. 2005; Rahmani, Alsahli, and Almatroodi 2017). A previous study revealed that pomegranates effectively reduce TNF‐α‐stimulated COX‐2 synthesis. According to a recent study, administration of a fruit extract has shown possible inhibitory effects on the development of cancer cells (Kim et al. 2013; Rahmani, Alsahli, and Almatroodi 2017; Berdowska, Matusiewicz, and Fecka 2021).

## 7 Invasion and Metastasis Inhibition

Cancer cells alter their biological pathways for invasion and metastasis. The most crucial stage of cancer development is metastasis, which determines treatment efficacy (Qian, Mei, and Zhang 2017). This evidence substantiates the notion that phosphoinositide 3‐kinase (PI3K), protein kinase B (Akt), and mammalian target of rapamycin (mTOR) signaling cascades facilitate metastasis. Numerous studies of prostate cancer, osteosarcomas, and glioblastomas have elucidated the pivotal involvement of this pathway in the propagation of cancerous cells (Zaino 2014). The biological phenomenon referred to as epithelial‐to‐mesenchymal transition (EMT) facilitates the conversion of epithelial cells into mesenchymal cells, thereby promoting the acquisition of malignancy in tumors. This alteration induces modifications in the functionality and morphology of the epithelial cells. This process is subject to modulation by many signaling pathways, including but not limited to TGF‐signaling, PI3K/Akt, NF‐κB, Wnt/β‐catenin, and Ras pathways (Xu, Yang, and Lu 2015). During this particular phase, there is an upregulation in the expression of N‐cadherin, SMA, Vimentin, and Fibronectin, accompanied by a downregulation in the levels of β‐catenin, E‐cadherin, CK8 and CK18 (Bonatti et al. 2018). Researchers have focused on this signaling system to reduce metastasis. This is because of the presence of PI3K in the ECM and the pre‐apoptotic mechanisms that induce apoptosis. Luteolin inhibits breast cancer metastatic processes, including angiogenesis, proliferation, and invasion (Franke et al. 2003). This mechanism of action is achieved through suppression of EMT, VEGF, apoptosis, and cellular proliferation. Numerous adverse consequences have been associated with diminished PI3K signaling. Luteolin effectively inhibited the molecular interactions between ERK and Ras, GSK3 and Akt, and Akt and PI3K, resulting in a notable reduction in the output of pathways involving these specific proteins. Breast carcinoma exhibits a noteworthy propensity for metastasis to the osseous structures. Metastatic breast cancer is a condition that unfortunately falls under incurable diseases, in cellular experiments involving MDA‐MB‐231 cells, the flavonoid known as p‐hydroxycinnamic acid exhibits notable anti‐metastatic properties. Cancerous cells require the presence of osteoclasts and osteoblasts, two distinct cellular entities responsible for the production of growth factors, to invade and permeate healthy bone tissues. Neoplastic cells must effectively persuade these cells to participate actively in invasion. However, in bone marrow co‐cultures with MDA‐MB231 cells, flavonoids inhibited these activities. The hypothesized effects of p‐hydroxycinnamic acid on the NF‐κB and PI3K signaling pathways may potentially alter the results mentioned above. Certain polyphenolic compounds found in pomegranates have been observed to exhibit the ability to attenuate Akt signaling pathways, concomitantly facilitating the elevation of miR‐101 expression levels. miR‐101 shows a distinct interaction pattern with TNM staging compared to Akt (Zhang et al. 2011).

## 8 Apoptosis Induction

**FIGURE 3:**
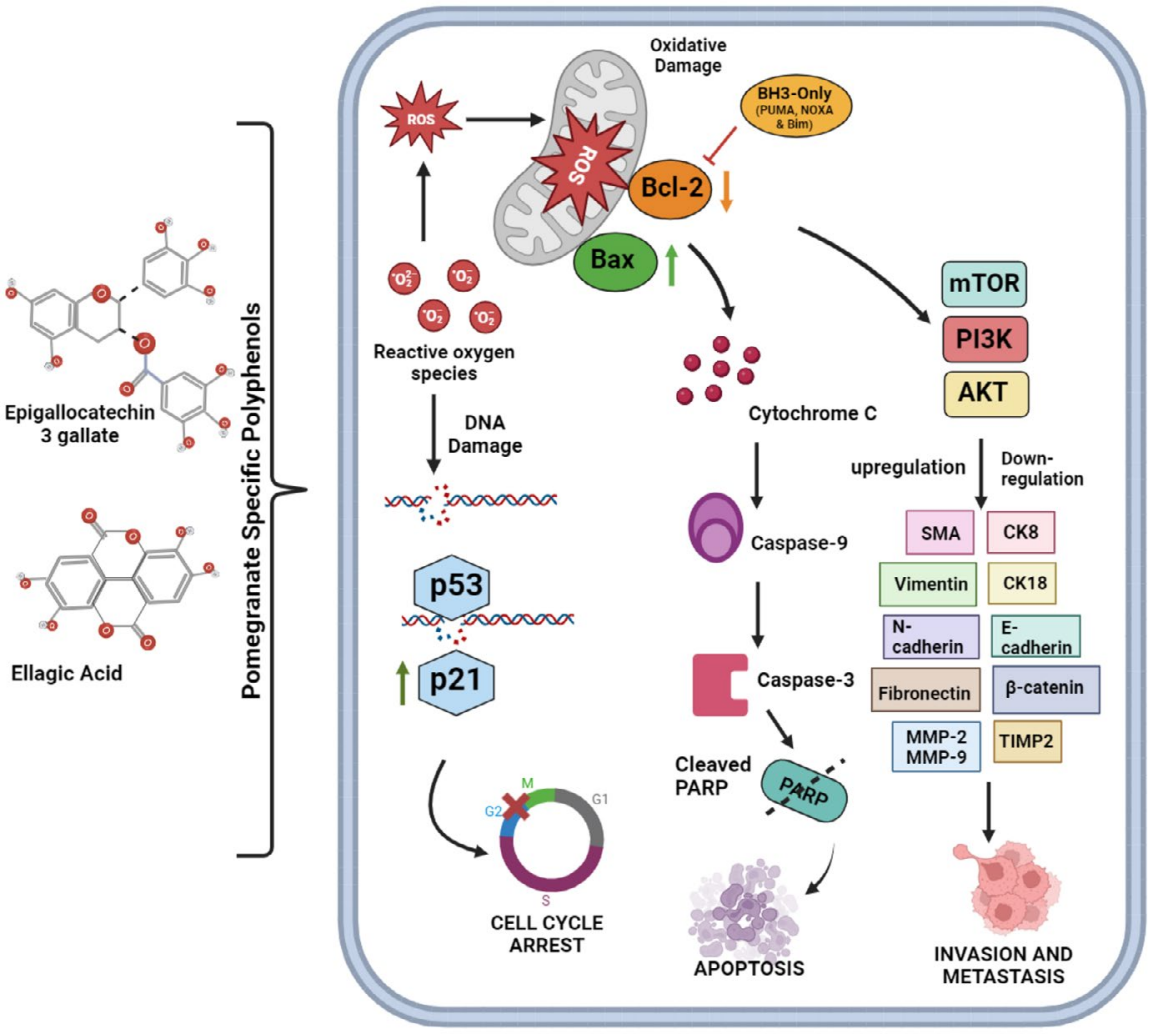
Cell cycle arrest, apoptosis and metastasis under the influence of pomegranate specific polyphenols.

Apoptosis is a cellular process employed to maintain the physiological and functional integrity of tissues. Apoptosis is a biological process triggered when cells experience significant and irreparable DNA damage. Cancerous cells can bypass apoptosis and remain in the body, which can become harmful over time. Malignant cells are characterized by mutations in the P53 gene and the downregulation of caspases, two apoptosis modulators (Wang 2016). Apoptosis can be triggered by the interaction of Bcl‐2 homology domain 3 (BH3)‐only proteins with pro‐apoptotic proteins such as p53 upregulated modulator of apoptosis (PUMA), NADPH oxidase activator (NOXA), and Bcl‐2‐interacting mediator of cell death (Bim). Epigallocatechin‐3 gallate, commonly referred to as EGCG, has been observed to elicit the process of programmed cell death, known as apoptosis, in various types of tumor cells, including those present in bladder, breast, prostate, and nasopharyngeal carcinomas. This was achieved by inhibiting Bcl‐2 protein synthesis and promoting Bax synthesis (Figure 3). The tumor suppressor, p53, modulates the cell cycle, induces cell cycle checkpoints, initiates apoptosis, repairs DNA, and inhibits tumor cell proliferation. Proapoptotic Bax is a downstream target of p35. Antineoplastic phytochemicals are primarily responsible for inducing apoptosis in breast and prostate carcinoma cells. This process involves preserving and enhancing p53 functionality, which is typically achieved by inhibiting miRNAs (miR‐34a) with tumor‐suppressive properties. In the realm of clinical research, a noteworthy revelation has come to light, indicating that a viable approach to combating cancer lies in directing our attention toward apoptosis as the principal target for intervention. One method for selectively inducing apoptosis involves attention toward peripheral pathways that are intricately involved in the regulation of cell survival and death. The pathways mentioned earlier encompass Akt and its subsequent signaling cascade, commonly called the P13K–Akt‐FOxO axis (Tung et al. 2013; Zhang et al. 2015). Polyphenols have the potential to modulate apoptosis by targeting this pathway (Aqil et al. 2012). Phytochemicals trigger two types of apoptosis, caspase‐dependent and caspase‐independent. Punicalagin, a pomegranate phytochemical, boosts caspase 3 and 9 activity to induce apoptosis in small cell lung cancer and human colorectal lymph nodes (Fjaeraa and Nånberg 2009; Koyama et al. 2010). Cancer may be linked to changes in the expression of Pro‐apoptotic Bax and anti‐apoptotic Bcl‐2 proteins. Ellagic acid enhances cell separation, decreases cell viability, and causes apoptosis in human neuroblastoma cells (Bishayee et al. 2016). Based on the observations made in the study conducted by Koyama et al., pomegranate extract inhibited cellular growth (Koyama et al. 2010). It induces apoptosis in prostate cancer cells during treatment (Jeune, Kumi‐Diaka, and Brown 2005). Bishayee and coworkers conducted a study to examine the chemo‐preventive potential of a pomegranate emulsion in the context of mammary tumorigenesis. According to their study, the emulsion inhibited breast cancer cell proliferation and induced apoptosis (Bhatia et al. 2013). Previous studies linked genistein and pomegranate extracts to MCF‐7 cell death. These drugs can be used as monotherapy or in combination regimens (Asmaa et al. 2015). Bioactive chemicals in pomegranate can restrict cellular proliferation by modulating the cell cycle and initiating apoptosis (Kiraz et al. 2016).

## 9 Cell Cycle Arrest Induction

The intricate cellular phenomena encompassed within a cellular entity, ultimately leading to the division and replication of said entity, usually known as the “cell cycle.” The G1 phase, colloquially referred to as the gap phase, the S phase denoting synthesis, the G2 phase representing interphase, and the M phase signifying mitosis encompass the four discernible stages constituting this intricate biological progression. Cell cycle arrest is a pivotal step in the development of cancer. Multiple studies have elucidated the significant role of pomegranates and their constituents in modulating cell cycle arrest, specifically in the G2/M phase of the cell cycle (Figure 3). The experimental phenomenon entails the discernment that the extract derived from the exocarp of the *Punica granatum* fruit exhibits a notable influence on suppressing cellular proliferation in K562 cells (Subkorn et al. 2021). This effect is primarily attributed to the arrest of cell cycle progression, specifically during the G2/M phase (Li et al. 2016). The scientific community has successfully identified multiple crucial checkpoints to accurately verify the completion of each stage of the cell cycle before progression. Numerous studies have revealed that the administration of pomegranate extract decelerates the progression of the G0/G1 phase of the cell cycle. Additionally, it inhibits the proliferation of a mouse breast cancer cell line (WA4). Prior investigations have postulated various pathways underlying these effects, including modifications in cellular signaling molecules and the machinery governing the cell cycle. In conjunction with its metabolite urolithin, ellagitannin reduced HT‐29 cell proliferation and clonogenic efficiency in dose‐ and time‐dependent manners. This process occurs during the G0/G1 and G2/M phases of cell replication. The underlying mechanism involves the induction of cell cycle arrest, which subsequently leads to the initiation of apoptosis. Flow cytometric analysis revealed that the botanical extract inhibited cell cycle progression, thereby inducing apoptosis and ultimately affecting the development of H1299 cells (Amin et al. 2009). The cytotoxic and apoptotic effects of pomegranate extracts have been evaluated in multiple myeloma cells and revealed significant pharmacological activity. The observed outcomes were achieved via modulation of mitochondrial membrane potential and initiation of cell cycle arrest (Li et al. 2016). In the DNA cell cycle assay, pomegranate therapy arrested the lung cancer cell cycle. The cell cycle is disrupted during the G0/G1 phase. The observed effect of cell arrest was directly correlated with the administered dosage. Pretreatment with pomegranate has been observed to enhances UVA‐induced cell cycle arrest, specifically in the G1 phase of normal human epidermal keratinocytes (NHEK).

Ellagic acid, a phenolic molecule, has demonstrated potent cytotoxicity against T24 human bladder cancer cells in vitro (Ceci et al. 2016). Studies of ellagic acid have revealed this phenomenon. The cell cycle can be effectively arrested in the G0/G1 phase by modulating the key molecular factors. This entails the upregulation of p53 and p21 expression, which play crucial roles in regulating cell division, while concurrently downregulating the expression of CDK2, which promotes cell cycle progression. During the G1‐S phase restriction point, the cell cycle and its progression are regulated by CDK4, which plays a pivotal role in orchestrating cellular development. For all of its suppression, the tumor suppressor p16^Ink4a^ targets CDK4, an essential CDK. Given the negligible significance of the N‐terminal region in the complete molecular structure, it has been demonstrated that the N‐terminal region of certain truncated p16^Ink4a^ molecules does not influence their interactions with CDK4. Nevertheless, McConnell et al. (1999) demonstrated that the C‐terminal region of p16^Ink4a^ is capable of inducing cell cycle arrest, impeding cellular growth, and facilitating interactions with CDK4/6.

## 10 Conclusion and Future Perspectives

Dietary nutrients are readily accessible and can potentially prevent and decelerate carcinogenesis, while maintaining a cost‐effective approach. The field of cancer therapy has garnered significant interest from both primary and clinical biologists, as well as from the general public, owing to its captivating nature. The primary hurdles lie in identifying the fundamental constituents of nutritional supplements that confer therapeutic benefits and elucidating the mechanisms by which they exert inhibitory effects on cancer. Continuing research is underway to explore the prospective antineoplastic attributes of the chemical constituents of pomegranates. Pomegranate fruit, including its juice and oil, has been proposed as a potential agent for chemoprevention and chemotherapy because of its ability to modulate signal transduction pathways. This modulation subsequently leads to notable anti‐inflammatory, anti‐proliferative, and anti‐tumorigenic effects. It is imperative to perform both in vitro and in vivo studies to ascertain potential interactions between pomegranate and other substances. These research methodologies determine whether the effects of pomegranate are complementary, synergistic, or antagonistic when combined with those of other compounds. Additionally, it is imperative to conduct human clinical trials to assess the antineoplastic efficacy of this compound in vitro and in vivo.

Preliminary evidence from animal and laboratory studies suggests that pomegranate exhibits potential anti‐cancer properties by inhibiting cancer growth. The constituents of pomegranate exhibit cytotoxic and anti‐proliferative properties against cancerous cells. In murine models, pomegranate‐derived products showed notable inhibitory effects on xenografted or chemically induced tumor proliferation. The phytochemicals found in pomegranates exhibit immunomodulatory, anti‐inflammatory, anti‐invasive, antioxidant, and antiangiogenic properties. Additionally, it is worth noting that they can modulate oncosuppressive signaling pathways. Based on the above research, it has been suggested that the phytochemical constituents found in pomegranates can impede the proliferation of neoplastic cells and ameliorate the development and progression of tumors. The phytochemicals in pomegranate exhibit a synergistic effect, not only among themselves but also among other phytochemicals and chemotherapeutic medications. This collective action results in enhanced anti‐cancer activity, surpassing the efficacy of individual molecules. These findings strongly suggest that pomegranate is a useful adjuvant chemotherapeutic agent. Oral administration of pomegranate extract increased the concentration of its active metabolite in specific organs affected by colorectal and prostate cancers, as evidenced by the findings of a clinical investigation. Preclinical results were substantiated by the administration of the oral pomegranate extract, demonstrating its ability to modulate cancer‐associated molecular markers. Multiple randomized controlled trials have provided evidence suggesting that the therapeutic efficacy of pomegranate is not significantly superior to that of placebo. Subsequent subgroup analyses revealed that pomegranate administration may provide therapeutic benefits to specific individuals with cancer. Additional in vivo and clinical data are required for drawing definitive conclusions. Research flaws have also been observed in the pomegranates. The absorption of the phytochemicals found in pomegranate may interfere with the efficacy of specific treatments. Based on the available clinical studies, it has been observed that the administration of supplements may lead to a decrease in the bioavailability of pomegranate phytochemicals. This finding helps to elucidate the differences observed between preclinical and clinical outcomes. Furthermore, it is worth noting that a significant proportion of in vitro investigations have primarily focused on examining breast, prostate, and gastrointestinal cancer cells. This observation underscores the necessity for further exploration of the potential anti‐cancer properties of pomegranate and other related cancer research areas. Several clinical studies exhibit limited sample sizes and focus on cancer objectives, while numerous preclinical findings pertaining to mechanisms and anti‐cancer advantages have yet to be replicated. Despite these inherent drawbacks, pomegranate shows immense potential as a chemopreventive and chemotherapeutic agent. The in vivo and in vitro testing results provided evidence supporting the safety profile of the pomegranate products. This study demonstrated the potential of pomegranates in enhancing the effectiveness of conventional chemotherapy while mitigating its adverse effects. Further investigation is strongly encouraged to elucidate additional molecular targets of pomegranate with anti‐cancer properties to provide a comprehensive understanding of its therapeutic benefits against cancer. It is imperative to explore the synergistic effects of the phytochemicals present in pomegranate that contribute to its anti‐cancer activity. In addition, a comparative analysis of the effects of pomegranate on various organ systems would be valuable. Finally, conducting randomized clinical trials with larger sample sizes would enhance the validity and reliability of our findings. Furthermore, it is imperative to implement altered pharmaceutical administration techniques, such as liposomes, nanoemulsions, nanoparticles, and innovative formulations to optimize the efficiency of phytochemical absorption. Based on the observations and analyses conducted in this study, it is evident that the constituents of pomegranates have promising potential as agents for chemo‐prevention and chemo‐therapeutic interventions. To acquire a holistic understanding of the therapeutic attributes of this intricate fruit in the context of cancer mitigation, future researchers must build on previous experimental endeavors.

## Author Contributions

**Abdur Rauf:** conceptualization (equal), writing – original draft (equal). **Ahmed Olatunde:** conceptualization (equal), writing – original draft (equal). **Zuneera Akram:** conceptualization (equal), writing – original draft (equal). **Hassan A. Hemeg:** methodology (equal), validation (equal). **Abdullah S. M. Aljohani:** methodology (equal), validation (equal), writing – review and editing (equal). **Waleed Al Abdulmonem:** conceptualization (equal), software (equal). **Ahood Khalid:** software (equal), validation (equal), visualization (equal). **Anees Ahmed Khalil:** visualization (equal), writing – original draft (equal). **Md. Rezaul Islam:** conceptualization (equal), software (equal), writing – review and editing (equal). **Rekha Thiruvengadam:** visualization (equal), writing – review and editing (equal). **Seung‐Hyun Kim:** software (equal), writing – review and editing (equal). **Muthu Thiruvengadam:** investigation (equal), supervision (equal), writing – review and editing (equal).

## Conflicts of Interest

The authors declare no conflicts of interest.

## Data Availability

The authors declare no conflicts of interest.
